# A new crystal phase of *N*,*N*,*N*′,*N*′-tetra­phenyl-1,1′-biphenyl-4,4′-diamine

**DOI:** 10.1107/S1600536809016389

**Published:** 2009-05-07

**Authors:** Xiangfeng Shao, Kenshiro Asahi, Takayoshi Yamauchi, Toyonari Sugimoto, Motoo Shiro

**Affiliations:** a Department of Chemistry, Graduate School of Science, Osaka Prefecture University, Sakai, Osaka 599-8570, Japan; b Rigaku Co., Akishima, Tokyo 196-8666, Japan

## Abstract

The complete molecule of the title compound, C_36_H_28_N_2_, is generated by a crystallographic centre of inversion. The biphenyl unit is forced by symmetry to be essentially flat (r.m.s. deviation = 0.008 Å); the dihedral angles between it and the two terminal phenyl rings are 69.39 (5) and 59.53 (5)°.

## Related literature

For the electronic properties of semiconductors based triaryl­amines, see, for example: Kennedy *et al.* (2002 ▶); Shirota (2000 ▶, 2005 ▶); Song *et al.* (2006 ▶). For the preparation of triaryl­amine, see: Hartwig (1999 ▶). For related structures, see: Kennedy *et al.* (2002 ▶); Low *et al.* (2004 ▶); Zhang *et al.* (2004 ▶, 2006 ▶).
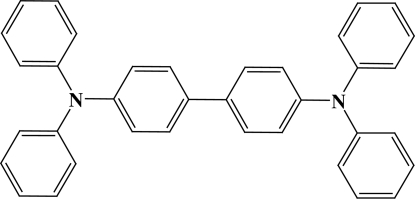


## Experimental

### 

#### Crystal data

C_36_H_28_N_2_
*M* *_r_* = 488.63
Monoclinic, 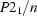

*a* = 9.6846 (2) Å
*b* = 14.2661 (4) Å
*c* = 9.7946 (2) Å
β = 107.0521 (15)°
*V* = 1293.75 (5) Å^3^
*Z* = 2
Cu *K*α radiation
μ = 0.56 mm^−1^
*T* = 173 K
0.15 × 0.14 × 0.11 mm

#### Data collection

Rigaku R-AXIS RAPID diffractometer
Absorption correction: multi-scan (*ABSCOR*; Higashi, 1995 ▶) *T*_min_ = 0.885, *T*_max_ = 0.941
14609 measured reflections
2362 independent reflections
1956 reflections with *F* ^2^ > 2σ(*F* ^2^)
*R*_int_ = 0.045

#### Refinement

*R*[*F* ^2^ > 2σ(*F* ^2^)] = 0.039
*wR*(*F* ^2^) = 0.119
*S* = 1.07
2362 reflections
174 parameters
H-atom parameters constrained
Δρ_max_ = 0.22 e Å^−3^
Δρ_min_ = −0.15 e Å^−3^

### 

Data collection: *PROCESS-AUTO* (Rigaku, 1998 ▶); cell refinement: *PROCESS-AUTO*; data reduction: *CrystalStructure* (Rigaku/MSC, 2007 ▶); program(s) used to solve structure: *SHELXS97* (Sheldrick, 2008 ▶); program(s) used to refine structure: *SHELXL97* (Sheldrick, 2008 ▶); molecular graphics: *ORTEPII* (Johnson, 1976 ▶); software used to prepare material for publication: *CrystalStructure*.

## Supplementary Material

Crystal structure: contains datablocks global, I. DOI: 10.1107/S1600536809016389/bq2132sup1.cif

Structure factors: contains datablocks I. DOI: 10.1107/S1600536809016389/bq2132Isup2.hkl

Additional supplementary materials:  crystallographic information; 3D view; checkCIF report

## Figures and Tables

**Fig. 1.**
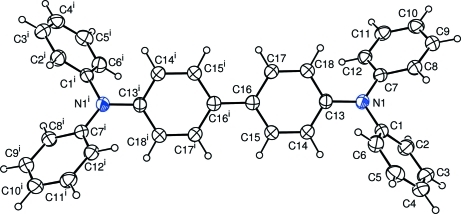
The molecular structure of (I), showing 50% probability displacement ellipsoids. [Symmetry code: (i) 1 - x, 1 - y, -z.]

**Fig. 2.**
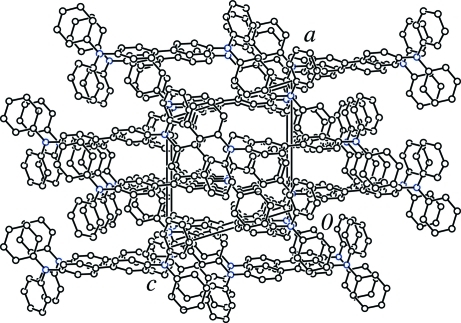
A packing diagram for (I), viewed along the b-axis. H atoms have been omitted.

## supplementary crystallographic information

### Comment

Triarylamine-based organic semiconductors have been intensively investigated as hole transport materials for electro-optic devices (Kennedy *et al.*, 2002; Shirota, 2000; Shirota, 2005). Recently, the organic field-effect transistor (OFET) based on a cyclic triphenylamine has been reported, which shows the relatively good mobility and high on/off ratio (Song *et al.*, 2006). The title compound, *N*,*N*,*N'*,*N'*-tetraphenyl-1,1'-biphenyl-4,4'-diamine (I), has been synthesized by coupling of two triphenylamine molecules using a copper catalyst (Ullman coupling) and also using methanesulfonic acid as catalyst in the yield of 10% (Zhang *et al.*, 2006). We have synthesized compound (I) from the coupling of 4,4'-dibromobiphenyl and diphenylamine using a palladium catalyst in the yield of 71% (Hartwig, 1999). The crystal structure of compound (I) has been reported (Zhang *et al.*, 2006), where the biphenyl part of the molecule is highly twisted with a dihedral angle of 32.9 (4)°. Herein, we report another crystal phase of compound (I).

The molecule (I) possesses a inversion center, thus the biphenyl moiety of the molecule shows a completely flat conformation with an average deviation of 0.0079Å (Fig. 1). The other fused rings make 69.39 (5)° and 59.53 (5)° dihedral angles with this flat biphenyl moiety. The N atom displays an almost trigonal geometry with slight distortion of the N—C bond lengths and C—N—C bond angles. The angle sums around the N atom is *ca* 360°. The molecular geometry of (I) is quite different from the previously reported one (Zhang *et al.*, 2006), and also different from those of the related biphenyl-diamine compounds (Kennedy *et al.*, 2002, Low *et al.*, 2004; Zhang *et al.*, 2004). The molecular packing pattern in the present crystal is also different from the reported one (Zhang *et al.*, 2006), and a herringbone-like packing motif governed by the van der Waals interactions is observed (Fig. 2).

### Experimental

A suspension of 4,4'-dibromobiphenyl (3.1 g, 10 mmol), diphenylamine (3.9 g, 21 mmol) and sodium *t*-butoxide (2.3 g, 24 mmol) in xylene (20 ml) was refluxed in the presence of palladium(II) acetate (4.4 mg, 0.020 mmol) and triphenylphosphine (42 mg, 0.016 mmol) for 5 h under an argon atmosphere. After removing the solvent in vacuum, the residue was separated by column chromatography on silica gel using benzene as eluent to afford the title compound in the yield of 71%. The single crystals of (I) suitable for the X-ray crystallographic analysis were obtained by recrystallization from benzene/hexane as colorless blocks.

### Refinement

All of the H atoms were positioned geometrically with C–H of 0.95Å and were constrained in a riding motion on their parent carbon atoms with *U*_iso_(H) = 1.2 *U*_eq_(C).

### Crystal data

**Table:**

C_36_H_28_N_2_	*F*(000) = 516.00
*M**_r_* = 488.63	*D*_x_ = 1.254 Mg m^−^^3^
Monoclinic, *P*2_1_/*n*	Melting point: 400 K
Hall symbol: -P 2yn	Cu *K*α radiation, λ = 1.54187 Å
*a* = 9.6846 (2) Å	Cell parameters from 9402 reflections
*b* = 14.2661 (4) Å	θ = 4.7–68.2°
*c* = 9.7946 (2) Å	µ = 0.56 mm^−^^1^
β = 107.0521 (15)°	*T* = 173 K
*V* = 1293.75 (5) Å^3^	Block, colorless
*Z* = 2	0.15 × 0.14 × 0.11 mm

### Data collection

**Table:**

Rigaku R-AXIS RAPID diffractometer	1956 reflections with *F*^2^ > 2σ(*F*^2^)
Detector resolution: 10.00 pixels mm^-1^	*R*_int_ = 0.045
ω scans	θ_max_ = 68.2°
Absorption correction: multi-scan (*ABSCOR*; Higashi, 1995)	*h* = −11→11
*T*_min_ = 0.885, *T*_max_ = 0.941	*k* = −17→16
14609 measured reflections	*l* = −11→11
2362 independent reflections	

### Refinement

**Table:**

Refinement on *F*^2^	H-atom parameters constrained
*R*[*F*^2^ > 2σ(*F*^2^)] = 0.039	*w* = 1/[σ^2^(*F*_o_^2^) + (0.0708*P*)^2^ + 0.1003*P*] where *P* = (*F*_o_^2^ + 2*F*_c_^2^)/3
*wR*(*F*^2^) = 0.119	(Δ/σ)_max_ = 0.001
*S* = 1.07	Δρ_max_ = 0.22 e Å^−^^3^
2362 reflections	Δρ_min_ = −0.15 e Å^−^^3^
174 parameters	Extinction correction: *SHELXL97* (Sheldrick, 2008)
0 restraints	Extinction coefficient: 0.0044 (7)

### Special details

**Table:**

Geometry. ENTER SPECIAL DETAILS OF THE MOLECULAR GEOMETRY
Refinement. Refinement was performed using all reflections. The weighted *R*-factor (*wR*) and goodness of fit (*S*) are based on *F*^2^. *R*-factor (gt) are based on *F*. The threshold expression of *F*^2^ > 2.0 σ(*F*^2^) is used only for calculating *R*-factor (gt).

### Fractional atomic coordinates and isotropic or equivalent isotropic displacement parameters (Å^2^)

**Table:**

	*x*	*y*	*z*	*U*_iso_*/*U*_eq_	
N1	0.61552 (11)	0.35882 (8)	0.48812 (11)	0.0350 (3)	
C1	0.75255 (14)	0.38277 (9)	0.58558 (13)	0.0322 (3)	
C2	0.76352 (16)	0.41058 (10)	0.72442 (14)	0.0407 (3)	
C3	0.89748 (16)	0.43064 (10)	0.81905 (15)	0.0460 (4)	
C4	1.02051 (16)	0.42680 (10)	0.77599 (16)	0.0435 (3)	
C5	1.00977 (15)	0.40187 (10)	0.63705 (16)	0.0419 (3)	
C6	0.87666 (15)	0.37871 (9)	0.54238 (15)	0.0375 (3)	
C7	0.52193 (14)	0.29751 (9)	0.53277 (13)	0.0312 (3)	
C8	0.57988 (15)	0.22955 (9)	0.63602 (13)	0.0364 (3)	
C9	0.49026 (15)	0.17243 (10)	0.68654 (14)	0.0408 (3)	
C10	0.34163 (15)	0.17989 (10)	0.63263 (14)	0.0406 (3)	
C11	0.28345 (15)	0.24546 (10)	0.52752 (14)	0.0370 (3)	
C12	0.37225 (14)	0.30446 (9)	0.47834 (13)	0.0329 (3)	
C13	0.57704 (13)	0.39859 (9)	0.34918 (13)	0.0303 (3)	
C14	0.61838 (14)	0.48974 (9)	0.33017 (13)	0.0333 (3)	
C15	0.58792 (14)	0.52884 (9)	0.19522 (13)	0.0322 (3)	
C16	0.51417 (12)	0.47929 (9)	0.07275 (12)	0.0286 (3)	
C17	0.46985 (14)	0.38858 (9)	0.09424 (13)	0.0338 (3)	
C18	0.50099 (14)	0.34835 (9)	0.22831 (13)	0.0344 (3)	
H2	0.6790	0.4158	0.7544	0.049*	
H3	0.9045	0.4473	0.9148	0.055*	
H4	1.1119	0.4412	0.8413	0.052*	
H5	1.0939	0.4006	0.6060	0.050*	
H6	0.8706	0.3600	0.4477	0.045*	
H8	0.6817	0.2224	0.6719	0.044*	
H9	0.5311	0.1277	0.7589	0.049*	
H10	0.2804	0.1405	0.6674	0.049*	
H11	0.1816	0.2501	0.4886	0.044*	
H12	0.3308	0.3498	0.4072	0.039*	
H14	0.6684	0.5258	0.4111	0.040*	
H15	0.6181	0.5912	0.1857	0.039*	
H17	0.4164	0.3533	0.0138	0.041*	
H18	0.4704	0.2861	0.2382	0.041*	

### Atomic displacement parameters (Å^2^)

**Table:**

	*U*^11^	*U*^22^	*U*^33^	*U*^12^	*U*^13^	*U*^23^
N1	0.0320 (6)	0.0420 (6)	0.0280 (5)	−0.0078 (4)	0.0041 (4)	0.0056 (4)
C1	0.0307 (7)	0.0328 (7)	0.0304 (6)	−0.0044 (5)	0.0049 (5)	0.0059 (4)
C2	0.0374 (7)	0.0514 (8)	0.0347 (7)	−0.0094 (6)	0.0125 (6)	−0.0006 (6)
C3	0.0506 (9)	0.0517 (9)	0.0323 (7)	−0.0171 (7)	0.0066 (6)	−0.0038 (6)
C4	0.0349 (7)	0.0412 (8)	0.0461 (8)	−0.0087 (6)	−0.0010 (6)	0.0025 (6)
C5	0.0311 (7)	0.0400 (7)	0.0550 (9)	0.0004 (5)	0.0132 (6)	0.0030 (6)
C6	0.0370 (8)	0.0406 (7)	0.0343 (7)	0.0005 (5)	0.0097 (5)	0.0019 (5)
C7	0.0328 (7)	0.0333 (7)	0.0272 (6)	−0.0060 (5)	0.0083 (5)	−0.0003 (4)
C8	0.0328 (7)	0.0389 (7)	0.0342 (7)	−0.0043 (5)	0.0047 (5)	0.0034 (5)
C9	0.0468 (8)	0.0386 (7)	0.0339 (7)	−0.0088 (6)	0.0069 (6)	0.0057 (5)
C10	0.0450 (8)	0.0430 (8)	0.0365 (7)	−0.0138 (6)	0.0162 (6)	−0.0024 (5)
C11	0.0315 (7)	0.0451 (8)	0.0354 (7)	−0.0055 (5)	0.0112 (5)	−0.0064 (5)
C12	0.0342 (7)	0.0346 (7)	0.0295 (6)	−0.0010 (5)	0.0086 (5)	−0.0015 (5)
C13	0.0280 (6)	0.0353 (7)	0.0273 (6)	0.0001 (5)	0.0078 (5)	0.0038 (4)
C14	0.0353 (7)	0.0330 (7)	0.0290 (6)	−0.0020 (5)	0.0052 (5)	−0.0014 (5)
C15	0.0345 (7)	0.0287 (6)	0.0323 (6)	−0.0013 (5)	0.0081 (5)	0.0014 (4)
C16	0.0231 (6)	0.0322 (6)	0.0307 (6)	0.0034 (4)	0.0081 (5)	0.0015 (4)
C17	0.0346 (7)	0.0367 (7)	0.0284 (6)	−0.0051 (5)	0.0064 (5)	−0.0012 (5)
C18	0.0375 (7)	0.0324 (7)	0.0328 (6)	−0.0060 (5)	0.0096 (5)	0.0021 (5)

### Geometric parameters (Å, °)

**Table:**

N1—C1	1.4304 (14)	C15—C16	1.3951 (16)
N1—C7	1.4178 (18)	C16—C16^i^	1.4920 (16)
N1—C13	1.4196 (16)	C16—C17	1.3986 (18)
C1—C2	1.3904 (19)	C17—C18	1.3829 (17)
C1—C6	1.388 (2)	C2—H2	0.950
C2—C3	1.3852 (18)	C3—H3	0.950
C3—C4	1.377 (2)	C4—H4	0.950
C4—C5	1.381 (2)	C5—H5	0.950
C5—C6	1.3892 (17)	C6—H6	0.950
C7—C8	1.3946 (17)	C8—H8	0.950
C7—C12	1.3936 (17)	C9—H9	0.950
C8—C9	1.384 (2)	C10—H10	0.950
C9—C10	1.3843 (19)	C11—H11	0.950
C10—C11	1.3828 (18)	C12—H12	0.950
C11—C12	1.387 (2)	C14—H14	0.950
C13—C14	1.3891 (18)	C15—H15	0.950
C13—C18	1.3951 (16)	C17—H17	0.950
C14—C15	1.3844 (17)	C18—H18	0.950
			
C4···C9^ii^	3.528 (2)	C14···H5^vi^	3.095
C12···C14^iii^	3.4707 (18)	C14···H10^viii^	3.150
N1···H14^iii^	3.582	C14···H12^iii^	3.370
C1···H18^iv^	3.262	C15···H2^iii^	2.881
C2···H18^iv^	3.427	C15···H4^vi^	3.061
C3···H3^v^	3.252	C15···H5^vi^	3.283
C3···H9^ii^	3.045	C15···H10^viii^	3.111
C3···H18^iv^	3.319	C16···H2^iii^	3.235
C4···H3^v^	3.412	C16···H10^viii^	3.004
C4···H9^ii^	2.912	C17···H8^ix^	3.482
C4···H10^ii^	3.562	C17···H10^viii^	2.910
C4···H15^vi^	3.418	C17···H11^viii^	3.230
C4···H18^iv^	3.081	C18···H8^ix^	3.144
C5···H9^ii^	3.436	C18···H10^viii^	2.941
C5···H11^vii^	3.315	C18···H11^viii^	3.599
C5···H14^vi^	3.445	H2···H11^iv^	3.291
C5···H15^vi^	3.513	H2···H14^iii^	3.382
C5···H18^iv^	2.922	H2···H15^iii^	3.102
C6···H5^vi^	3.515	H3···H3^v^	2.581
C6···H9^viii^	3.520	H3···H4^v^	2.914
C6···H10^viii^	3.525	H3···H9^ii^	3.245
C6···H17^iv^	3.353	H3···H9^iv^	3.404
C6···H18^iv^	3.001	H3···H10^iv^	3.298
C7···H14^iii^	3.263	H4···H9^ii^	3.027
C8···H3^ix^	3.433	H4···H10^ii^	3.038
C8···H11^iv^	3.314	H4···H15^vi^	2.743
C8···H12^iv^	3.234	H4···H17^xiii^	3.194
C9···H3^x^	3.597	H4···H18^iv^	3.549
C9···H3^ix^	3.065	H5···H6^vi^	3.488
C9···H4^x^	3.432	H5···H9^viii^	3.298
C9···H6^xi^	3.137	H5···H11^vii^	2.687
C9···H11^iv^	3.192	H5···H12^vii^	3.493
C9···H12^iv^	3.387	H5···H14^vi^	2.580
C10···H3^ix^	2.996	H5···H15^vi^	2.935
C10···H4^x^	3.435	H5···H18^iv^	3.332
C10···H6^xi^	3.069	H6···H9^viii^	2.748
C11···H2^ix^	3.447	H6···H10^viii^	2.626
C11···H3^ix^	3.303	H6···H11^vii^	3.314
C11···H5^xii^	3.112	H6···H17^iv^	3.116
C11···H8^ix^	3.361	H6···H18^iv^	3.429
C11···H9^ix^	3.521	H8···H11^iv^	3.127
C11···H14^iii^	3.326	H8···H12^iv^	2.551
C11···H15^iii^	3.557	H8···H17^iv^	3.286
C12···H5^xii^	3.562	H8···H18^iv^	2.682
C12···H8^ix^	3.052	H9···H11^iv^	2.881
C12···H9^ix^	3.508	H9···H12^iv^	2.857
C12···H14^iii^	2.728	H10···H17^xi^	3.404
C12···H15^iii^	3.587	H10···H18^xi^	3.437
C13···H2^iii^	3.566	H11···H14^iii^	3.529
C13···H10^viii^	3.068	H11···H17^xi^	3.034
C14···H2^iii^	3.066	H12···H14^iii^	2.511
			
C1—N1—C7	119.56 (10)	C1—C2—H2	120.0
C1—N1—C13	118.40 (10)	C3—C2—H2	120.0
C7—N1—C13	122.03 (9)	C2—C3—H3	119.6
N1—C1—C2	120.71 (13)	C4—C3—H3	119.6
N1—C1—C6	120.24 (11)	C3—C4—H4	120.3
C2—C1—C6	119.05 (11)	C5—C4—H4	120.3
C1—C2—C3	120.09 (14)	C4—C5—H5	119.8
C2—C3—C4	120.78 (13)	C6—C5—H5	119.8
C3—C4—C5	119.37 (12)	C1—C6—H6	119.9
C4—C5—C6	120.41 (15)	C5—C6—H6	119.9
C1—C6—C5	120.25 (13)	C7—C8—H8	119.7
N1—C7—C8	119.66 (11)	C9—C8—H8	119.8
N1—C7—C12	121.70 (10)	C8—C9—H9	119.7
C8—C7—C12	118.63 (12)	C10—C9—H9	119.7
C7—C8—C9	120.50 (12)	C9—C10—H10	120.4
C8—C9—C10	120.63 (12)	C11—C10—H10	120.4
C9—C10—C11	119.15 (14)	C10—C11—H11	119.7
C10—C11—C12	120.69 (12)	C12—C11—H11	119.7
C7—C12—C11	120.36 (11)	C7—C12—H12	119.8
N1—C13—C14	119.85 (10)	C11—C12—H12	119.8
N1—C13—C18	122.21 (11)	C13—C14—H14	119.5
C14—C13—C18	117.93 (11)	C15—C14—H14	119.5
C13—C14—C15	121.07 (10)	C14—C15—H15	119.0
C14—C15—C16	121.92 (11)	C16—C15—H15	119.0
C15—C16—C16^i^	121.67 (11)	C16—C17—H17	118.8
C15—C16—C17	116.22 (10)	C18—C17—H17	118.8
C16^i^—C16—C17	122.11 (10)	C13—C18—H18	119.8
C16—C17—C18	122.40 (10)	C17—C18—H18	119.8
C13—C18—C17	120.43 (11)		
			
C(1)—N(1)—C(7)—C(8)	−29.27 (18)	N(1)—C(7)—C(8)—C(9)	176.51 (12)
C(1)—N(1)—C(7)—C(12)	149.31 (12)	N(1)—C(7)—C(12)—C(11)	−177.89 (12)
C(7)—N(1)—C(1)—C(2)	−47.50 (17)	C(8)—C(7)—C(12)—C(11)	0.71 (19)
C(7)—N(1)—C(1)—C(6)	132.65 (13)	C(12)—C(7)—C(8)—C(9)	−2.12 (19)
C(1)—N(1)—C(13)—C(14)	−35.87 (18)	C(7)—C(8)—C(9)—C(10)	1.9 (2)
C(1)—N(1)—C(13)—C(18)	142.58 (13)	C(8)—C(9)—C(10)—C(11)	−0.1 (2)
C(13)—N(1)—C(1)—C(2)	131.78 (13)	C(9)—C(10)—C(11)—C(12)	−1.3 (2)
C(13)—N(1)—C(1)—C(6)	−48.07 (17)	C(10)—C(11)—C(12)—C(7)	1.0 (2)
C(7)—N(1)—C(13)—C(14)	143.40 (13)	N(1)—C(13)—C(14)—C(15)	177.10 (12)
C(7)—N(1)—C(13)—C(18)	−38.16 (19)	N(1)—C(13)—C(18)—C(17)	−177.79 (12)
C(13)—N(1)—C(7)—C(8)	151.47 (12)	C(14)—C(13)—C(18)—C(17)	0.7 (2)
C(13)—N(1)—C(7)—C(12)	−29.94 (18)	C(18)—C(13)—C(14)—C(15)	−1.4 (2)
N(1)—C(1)—C(2)—C(3)	177.90 (12)	C(13)—C(14)—C(15)—C(16)	0.4 (2)
N(1)—C(1)—C(6)—C(5)	−179.93 (12)	C(14)—C(15)—C(16)—C(17)	1.39 (19)
C(2)—C(1)—C(6)—C(5)	0.21 (19)	C(14)—C(15)—C(16)—C(16)^i^	−178.00 (12)
C(6)—C(1)—C(2)—C(3)	−2.2 (2)	C(15)—C(16)—C(16)^i^—C(17)^i^	−0.65 (19)
C(1)—C(2)—C(3)—C(4)	2.4 (2)	C(15)—C(16)—C(17)—C(18)	−2.1 (2)
C(2)—C(3)—C(4)—C(5)	−0.5 (2)	C(16)^i^—C(16)—C(17)—C(18)	177.25 (12)
C(3)—C(4)—C(5)—C(6)	−1.5 (2)	C(17)—C(16)—C(16)^i^—C(15)^i^	0.65 (19)
C(4)—C(5)—C(6)—C(1)	1.7 (2)	C(16)—C(17)—C(18)—C(13)	1.1 (2)

Symmetry codes: (i) −*x*+1, −*y*+1, −*z*; (ii) −*x*+3/2, *y*+1/2, −*z*+3/2; (iii) −*x*+1, −*y*+1, −*z*+1; (iv) *x*+1/2, −*y*+1/2, *z*+1/2; (v) −*x*+2, −*y*+1, −*z*+2; (vi) −*x*+2, −*y*+1, −*z*+1; (vii) *x*+1, *y*, *z*; (viii) *x*+1/2, −*y*+1/2, *z*−1/2; (ix) *x*−1/2, −*y*+1/2, *z*−1/2; (x) −*x*+3/2, *y*−1/2, −*z*+3/2; (xi) *x*−1/2, −*y*+1/2, *z*+1/2; (xii) *x*−1, *y*, *z*; (xiii) *x*+1, *y*, *z*+1.

## References

[bb1] Hartwig, J. F. (1999). *Pure Appl. Chem.* **71**, 1417–1423.

[bb2] Higashi, T. (1995). *ABSCOR* Rigaku Corporation, Tokyo, Japan.

[bb3] Johnson, C. K. (1976). *ORTEPII* Report ORNL-5138. Oak Ridge National Laboratory, Tennessee, USA.

[bb4] Kennedy, A. R., Smith, W. E., Tackley, D. R., David, W. I. F., Shankland, K., Brown, D. & Teat, S. J. (2002). *J. Mater. Chem.* **12**, 168–172.

[bb5] Low, P. J., Paterson, M. A. J., Puschmann, H., Goeta, A. E., Howard, J. A. K., Lambert, C., Cherryman, J. C., Tackley, D. R., Leeming, S. & Brown, B. (2004). *Chem. Eur. J.* **10**, 83–91. 10.1002/chem.200305200 14695553

[bb6] Rigaku (1998). *PROCESS-AUTO* Rigaku Corporation, Tokyo, Japan.

[bb7] Rigaku/MSC (2007). *CrystalStructure* Rigaku/MSC, The Woodlands, Texas, USA.

[bb8] Sheldrick, G. M. (2008). *Acta Cryst.* A**64**, 112–122. 10.1107/S0108767307043930 18156677

[bb9] Shirota, Y. (2000). *J. Mater. Chem.* **10**, 1–25.

[bb10] Shirota, Y. (2005). *J. Mater. Chem.* **15**, 75–93.

[bb11] Song, Y., Di, C., Yang, X., Li, S., Xu, W., Liu, Y., Yang, L., Shuai, Z., Zhang, D. & Zhu, D. (2006). *J. Am. Chem. Soc.* **128**, 15940–15941. 10.1021/ja064726s 17165699

[bb12] Zhang, Z., Burkholder, E. & Zubieta, J. (2004). *Acta Cryst.* C**60**, o452–o454. 10.1107/S0108270104010479 15178877

[bb13] Zhang, H.-G., Yu, W.-T., Yan, S.-N., Cheng, C. & Tao, X.-T. (2006). *Acta Cryst.* E**62**, o5236–o5238.

